# Integrating drip fertigation and nitrification inhibitors for rhizosphere-scale control of nitrogen transformations

**DOI:** 10.3389/fpls.2026.1894587

**Published:** 2026-09-17

**Authors:** Muhammad Zain, Sheheryar Khan, Hongjun Lei, Abdul Ghafoor

**Affiliations:** 1 College of Water Conservancy, North China University of Water Resources and Electric Power, Zhengzhou, China; 2 Water and Environmental Studies Center, King Faisal University, Al-Ahsa, Saudi Arabia

**Keywords:** drip irrigation, nitrification inhibitor, nitrogen transformation, rhizosphere process, wetted bulb

## Abstract

Reactive nitrogen (Nr) losses from fertilized agricultural soils remain one of the dominant drivers of groundwater nitrate contamination and nitrous oxide (N_2_O) emissions, yet mitigation strategies are largely designed around input optimization rather than control of in-soil nitrogen transformations. Drip fertigation and nitrification inhibitors (NIs) have independently shown potential to improve nitrogen efficiency, however their integrated effects remain fragmented across irrigation and inhibitor-focused literature. Consequently, an integrated mechanistic framework explaining how drip irrigation-induced microenvironments regulate NI performance and nitrogen transformation is lacking. Here, we argue that the integration of drip fertigation with NIs, hereafter referred to as drip-NIs integration, is best understood as rhizosphere process control rather than input management: drip defines a bounded reaction-transport domain, and NIs slow down the conversion of ammonium (NH_4_^+^) to nitrate (NO_3_^-^). This review aims to synthesize evidence across NI types, crops, and drip configurations to examine how localized wetting patterns govern ammonium-nitrate partitioning, microbial processes, and inhibitor fate within the root zone. The available evidence suggests that NI performance may depend on spatial and temporal overlap among oxygen recovery, NH_4_^+^ availability, and active ammonia oxidizers within the wetted bulb, however direct spatial validation remains limited. Based on these insights, we propose the Designer Rhizosphere Model (DRM) as a conceptual, hypothesis-generating framework linking controllable design variables (emitter placement, irrigation waveform, fertigation chemistry, NI formulation) with the measurable state variables (oxygen availability, NH_4_^+^/NO_3_^-^ fields, inhibitor exposure), microbial process rates, and yield-scaled environmental outcomes. The DRM requires validation through spatially field experiments before practical application. The resulting framework suggests that drip-NIs integration may reduce nitrate leaching and greenhouse gas emissions while sustaining productivity, but outcomes are context-dependent and may be constrained by salinity accumulation, acidification, oxygen limitation, ammonium toxicity, and off-target effects. Future research should prioritize spatially resolved measurements, sensor-guided fertigation, drip-compatible inhibitor formulations, and process-based decision support models. Collectively, this review reframes nitrogen management from simple fertilizer placement to rhizosphere process control for improving agricultural sustainability.

## 1 Introduction

Modern agriculture is based on reactive nitrogen (Nr), yet it utilizes the nitrogen (N) with low efficiency. Across diverse cropping systems, only a portion of the applied nitrogen fertilizer is recovered in terms of harvested biomass, while the remainder may be retained in the soil or lost to the atmosphere and water bodies as reactive nitrogen forms, leading to a well-known nitrogen cascade: contamination of groundwater with nitrate (NO_3_^-^), eutrophication and nitrous oxide (N_2_O) emissions (Galloway et al., 2008; Zhang et al., 2015). Among these effects, N_2_O is especially significant, as it is not only a potent greenhouse gas but also the major ozone-depleting emission of this century (Ravishankara et al., 2009). The central challenge is not just how to minimize inputs, but how to retain nitrogen within the root-active zone in plant-available forms, at times when the roots are able to absorb it rather than microbes and transport pathways remove it.

This challenge of minimizing fertilizer input is now framed in the context of the Earth-system level. Recent assessments of the planetary boundaries framework identify biogeochemical flows, such as nitrogen, as one of the boundaries that has already been transgressed, indicating that the present patterns of nitrogen production and environmental loss exceed levels compatible with long-term stability of the Earth-system (Steffen et al., 2015; Richardson et al., 2023). Scenario analysis implies that, without an effective mitigation strategy, reactive nitrogen pollution is likely to intensify further amid increasing food demand (Bodirsky et al., 2014). Against this backdrop, enhancing nitrogen use efficiency (NUE) is not just a goal in agronomy, but also an almost non-negotiable requirement for sustainability. Practically, a substantial part of nitrogen management is structured around the 4R nutrient stewardship framework: right source, right rate, right time, and right place (Johnston and Bruulsema, 2014). The value of this framing is clear, although its implementation can be strengthened by more explicitly considering soil as an active biogeochemical system. In fact, soil is an active reactor in which physical transport, redox structure, and microbial metabolism determine nitrogen fate once applied to the soil (Stein and Klotz, 2016; Coskun et al., 2017). The main loss pathways such as nitrification and denitrification result from interactions among soil moisture and oxygen status, substrate supply, and microbial community structure (Butterbach-Bahl et al., 2013; Qin et al., 2021; Wu et al., 2023). Therefore, the practical implication is that “right place” should encompass not only fertilizer placement but also the regulation of in-soil nitrogen transformation within a structured soil microenvironment.

Drip irrigation presents an underused lever to this type of process control, since it is not simply a water-saving technology. Through the application of small and frequent doses of water to the root zone, drip irrigation forms a spatially confined wetted bulb, defined here as the three-dimensional soil volume wetted around an emitter, within which water content, oxygen availability, solute concentration, and root activity vary spatially and temporally (Al-Ogaidi et al., 2016; Rank et al., 2019). In high-value and water-limited systems, subsurface drip irrigation (SDI) has been widely adopted because it minimizes non-beneficial evaporation (Nalliah et al., 2009), and enables more precise fertigation; however, it also reshapes the soil habitat where nitrogen transformations take place (Ayars et al., 2015). From a biogeochemical perspective, the wetted bulb functions as a semi-engineered reactor; the emitter structure and irrigation schedule define the hydraulic template, which in turn regulates oxygen availability and solute transport, as well as the spatial distribution of microbial activity. Such a reproducible spatial structure creates an opening to treat nitrification as a controlled reaction occurring in predictable zones within the bulb, rather than a background loss process.

The second, independent line of innovation to reduce nitrogen losses is inhibition of nitrification, which is microbial oxidation of ammonium (NH_4_^+^) into NO_3_^-^. Nitrification inhibitors (NIs), which are used commercially, have consistently shown the possibility of enhancing NUE and minimizing environmental losses (Sha et al., 2020), but their performance is inconsistent across soils and climates. A broad meta-analysis reported average increases in crop yield and NUE with nitrification and urease inhibitors, and also highlighted a high level of dependence on soil texture, irrigation level, and management environment (Abalos et al., 2014). A complementary synthesis emphasizes that the sensitivity of NIs that causes its strong performance also renders it inconsistent because the activity of the inhibitors is temperature-dependent, moisture-dependent, and is influenced by the compatibility between place of occurrence of inhibitor and the site of active nitrification (Ruser and Schulz, 2015). However, NI responses are not uniformly beneficial. Under hot, dry conditions, 3,4-dimethylpyrazole phosphate (DMPP) and N-(n-butyl) thiophosphoric triamide (NBPT) did not increase irrigated pasture yield (Dougherty et al., 2016). Nauer et al. conducted a three-season study on Australian cropping and dairy farms and found no significant effects of DMPP-amended urea on crop and pasture yield (Nauer et al., 2018). Similarly, in another study, several NIs did not significantly affect the silage maize yield, N_2_O emissions, and nitrogen removal in a silty loam soil (Herr et al., 2020). In other words, NIs are not plug-in tools; they perform best when inhibitor molecules remain in effective contact with microsites where NH_4_^+^ and oxygen co-exist.

Many agronomic trials still use NIs by broadcast blending or banding without paying any particular attention to how irrigation structures the micro-environment or where nitrification is focused. In cases where the inhibitor is spatially separated from the hotspots of nitrification, or the wetting events are faster than the transport or degradation, the effective inhibitor-microbe contact may be brief, thus leading to inconsistent findings despite a good understanding of the chemistry. Therefore, the integration of drip fertigation with NIs, hereafter referred to as drip-NIs integration, could be a potential approach since it has the capability to repeatedly co-deliver NH_4_^+^ (or NH_4_^+^ derived from urea) and a nitrification inhibitor to the same confined volume of soil at controlled times. In principle, NI application under drip irrigation should enhance the likelihood that inhibitors coincide with active nitrification zones, and should allow irrigation scheduling to reinforce inhibitory conditions over time rather than after a single application pulse.

Our structured literature search identified studies that directly evaluated NIs under surface or subsurface drip irrigation. Although the reported results are generally encouraging, the evidence base remains limited and heterogenous across crops, experimental settings, inhibitor formulations, drip configurations, and measured outcomes. Combinations of urease-nitrification inhibitor formulations with drip fertigation have been found to lower cumulative greenhouse gas emissions and enhance nitrogen recovery compared to uninhibited fertigation in greenhouse production systems (Sunling et al., 2024). In a field-cropping context, a recent comparison of various nitrification inhibitors under subsurface banding and subsurface drip in wheat has demonstrated that the delivery pathway is as important as the inhibitor identity when the goal is to reduce emissions without causing any yield loss (Ribeiro et al., 2024). Other studies comparing surface and subsurface drip fertigation also indicate that the layout of drip systems can reshape both N_2_O emissions and the distribution of N-cycling functional genes, which supports the concept that the design of irrigation systems can reorganize functional habitats of nitrogen-transforming microbes (Guardia et al., 2024). Taken together, these studies suggest that drip systems may influence NI performance by modifying the rhizosphere conditions under which microbial nitrogen transformations occur; however, broader field validation across soils, crops, and climatic conditions are still required.

The literature, however, remains fragmented across disciplines. Existing studies of drip irrigation have mainly focused on crop water productivity, fertigation efficiency, and salt management, whereas NIs research has largely emphasized on inhibitor chemistry, N_2_O reduction, and agronomic responses. What remains lacking is an integrated rhizosphere framework that links drip-created wetting patterns with inhibitor persistence, placement, and microbial nitrification activity. Therefore, this review aims to (i) synthesize how drip irrigation structures the physical and chemical environment of the wetted bulb; (ii) evaluate how NI performance depends on microenvironmental conditions and spatiotemporal alignment with active nitrification; and (iii) propose the Designer Rhizosphere Model (DRM) as a hypothesis-generating framework connecting controllable management variables with measurable wetted-bulb conditions, and agronomic and environmental outcomes. This integration of drip irrigation and NIs, rather than a separate evaluation, constitutes the novelty of this review. Finally, we identify the modeling and measurement priorities required to test the framework and translate it into practical management.

## 2 Review methodology

This review followed a structured literature-search approach to examine how drip fertigation and nitrification inhibitors interact within the rhizosphere. Search engines and databases such as Web of Science, ScienceDirect, Google Scholar, Scopus, and PubMed were used to identify peer-reviewed scientific literature. The search covered publications available from database inception to December 2025.

The primary search combined terms related to localized irrigation: “drip irrigation” OR “drip fertigation” OR “subsurface drip irrigation” OR “localized irrigation” with terms related to nitrification inhibitors: “nitrification inhibitor” OR “dicyandiamide” OR “DCD” OR “3,4-dimethylpyrazole phosphate” OR “DMPP” OR “DMPSA” OR “dimethylpyrazole succinic acid” OR “nitrapyrin”. The terms related to nitrogen transformation or environmental outcomes were: “nitrification” OR “ammonia oxidation” OR “ammonia oxidizer” OR “nitrous oxide” OR “N_2_O” OR “nitrate leaching” OR “nitrogen use efficiency”. Terms within each group were combined using the Boolean operator OR, whereas the nitrification-inhibitor group, and the nitrogen-transformation or environmental-outcome group were combined using AND. Further, a keyword co-occurrence analysis was performed using VOSviewer, and the resulting network is presented in **Figure 1**.

**Figure 1:**
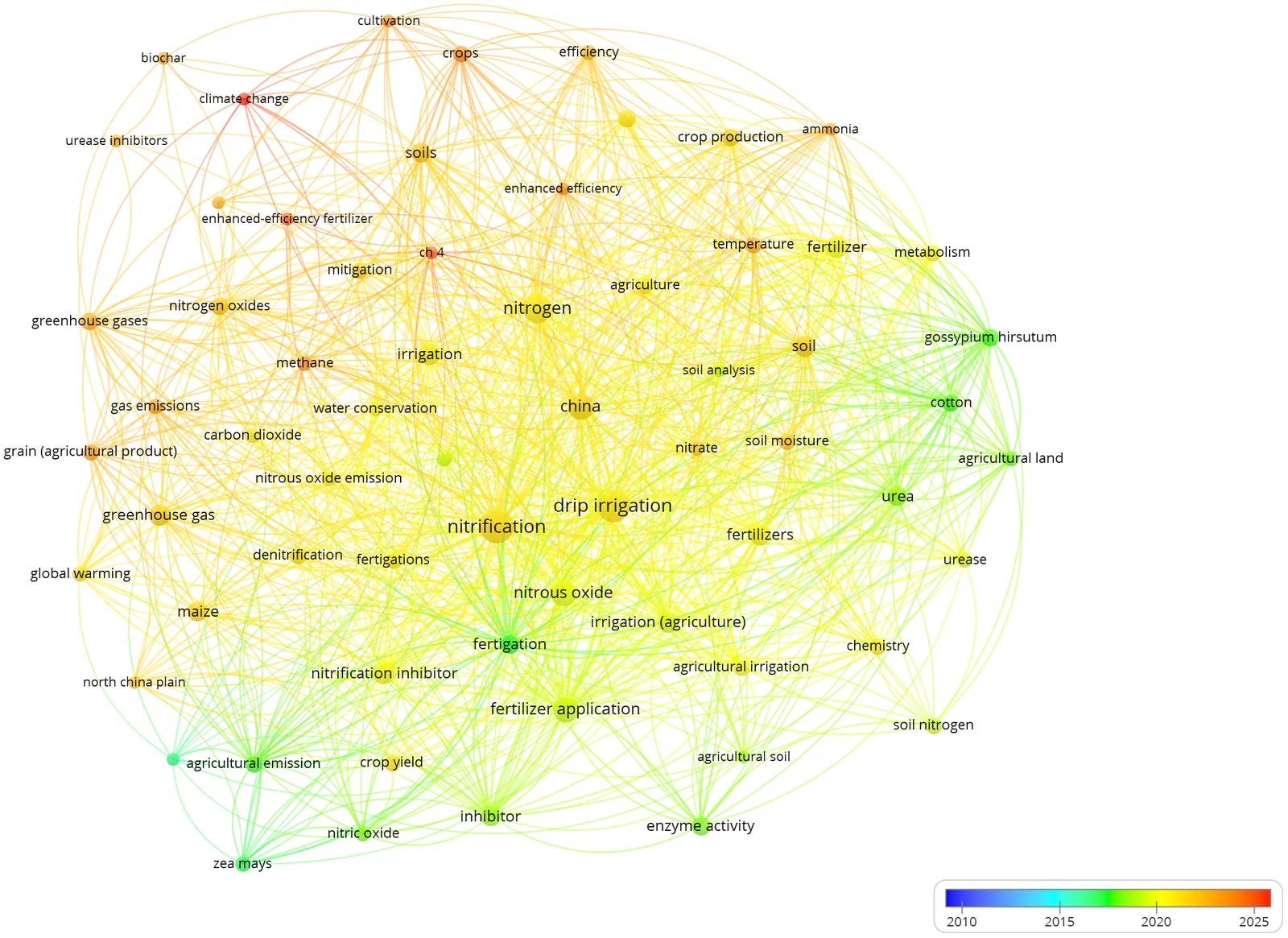
Co-occurrence network of keywords from studies on drip irrigation, fertigation, and nitrification inhibitors (NIs).

The article selection process was conducted in two steps: Initially, the titles and abstracts of retrieved articles were reviewed. In the second step, the selected articles were read in full and eligibility criteria were applied. Peer-reviewed studies conducted in agricultural soils were considered eligible. Primary emphasis was placed on field and greenhouse-grown studies evaluating nitrification inhibitors under drip irrigation systems. However, such studies remain limited; soil studies examining drip irrigation and mineral nitrogen distribution were also included. Studies were excluded if they were published in languages other than English, involved animal studies, case reports, conference proceedings, or editorials. Finally, we summarized the results and proposed the hypothesized DRM framework and derived future research perspectives from this analysis.

## 3 Drip-irrigated wetted bulb as a biogeochemical habitat

For conceptual purposes, the three-dimensional wetted bulb formed around a drip emitter can be described as three intergrading zones whose boundaries and physicochemical and biological properties vary with soil hydraulic characteristics, emitter configuration, irrigation management, and time after irrigation (**Table 1**). The emitter-proximal region may, under some soil and irrigation conditions, be characterized by temporary hypoxia and chemical reactions after irrigation, where NO_3_^-^ and readily available carbon may promote denitrification and associated N_2_O and N_2_ losses to the atmosphere. The peripheral wetted front, however, may remain too dry to support denitrification under some conditions. The wetted bulb is usually considered as a delivery footprint for water and nutrients. In practice, it is a hydraulically structured microenvironment that structures diffusion and advection, as well as persistence of solutes and gases. These physical controls in turn shape where nitrogen transformations occur and the strength of their responses to management. Classic drip hydraulics work had already framed the wetted soil volume as a context-dependent geometry governed by soil texture, emitter discharge and spacing (Schwartzman and Zur, 1986; Lazarovitch et al., 2007; Kilic, 2020). For nitrification control, the key step is to consider the wetted bulb as a designable biogeochemical habitat. Here, “habitat” is literal; the wetted bulb defines the overlap among water availability, oxygen diffusion, mineral-N substrates and root activity, which together determine the portion of the environment in which nitrifiers and denitrifiers can be active.

**Table 1:** Conceptual comparison of the physicochemical and biological characteristics of soil-water microenvironments across drip-irrigation wetting zones.

Characteristics	Near-emitter wet core	Intermediate wetted zone	Outer wetting front	Citation
Spatial position relative to emitter	The region immediately surrounding the emitter.	The wetted region between emitter proximal region and advancing wetting boundary.	The advancing boundary of the wetted volume, where recently wetted soil meets the comparatively drier soil.	Li et al., 2004b; Skaggs et al., 2010
Soil moisture status	Generally, has the highest soil moisture content immediately after irrigation.	Soil water content declines away from the source and changes with irrigation rate, volume, and soil properties.	Represents the transition from recently wetted soil toward the antecedent soil water condition.	Skaggs et al., 2010
Oxygen availability	Oxygen concentration may decrease after water application, particularly in intensively irrigated clayey soils.	Oxygen availability generally recovers as soil drains and dries.	The wetting front should not be assumed to be consistently fully aerated, because its oxygen status depends on soil texture, and time after irrigation.	Friedman and Naftaliev, 2012
pH response	Ammonium and urea-based fertigation may cause localized acidification.	The spatial extent of acidification depends on N form and emitter discharge.	No consistent alkalinization or acidification pattern has been established.	Haynes, 1990
Root distribution	Root density should not be assumed to be sparse solely because this region is wet.	In potato, higher irrigation frequency increased root-length density within 0–60 cm soil layer.	Roots were not confined to the wetted soil volume even under highest irrigation frequency.	Wang et al., 2006
Microbial community and activity	In one study, bacterial diversity was significantly lower near the emitter.	Experimental aeration increased soil, microbial respiration, and microbial abundance, showing oxygen availability can regulate biological activity.	Microbial communities sampled 45 cm laterally from subsurface emitters differed from those sampled within 15 cm.	Quach et al., 2022
Mineral-N distributions	Ammonium may accumulate close to the emitter. High NH_4_^+^ concentration were measured approximately 2.-7.5 cm from the source.	Urea and nitrate were more mobile than ammonium and moved vertically and laterally through the wetted profile.	Nitrate accumulation at the boundary of the wetted volume was observed in the point-source fertigation experiment.	Li et al., 2003
Nitrogen transformation	Large ammonium accumulation below the emitter may retard nitrification.	Studies do not establish universal spatial zone of maximum nitrification.	The available cited experiments do not support a universal claim that denitrification or another N process dominates at the outer wetting front.	Friedman and Naftaliev, 2012
Nutrient availability and crop nutrient uptake	High mineral-N concentration does not necessarily imply high uptake.	Aerated drip irrigation improved root-zone oxygen status and was associated with higher nitrogen uptake in tomato, but study did not identify a universal spatial zone of maximum uptake.	Because roots may extend beyond the visibly wetted bulb, crop uptake should not be described universally as negligible.	Zhu et al., 2022

Here, the terms are not universally standardized hydrological zones, and their spatial boundaries and characteristics may vary with soil properties, irrigation frequency and volume, emitter discharge, and time after irrigation.

### 3.1 Hydrologic and physical structure of the wetted bulb

Drip irrigation generates steep and reproducible water content gradients around an emitter. It has been shown through measurements and modeling that the resultant wetted bulb is characterized by a typical three-dimensional shape with the lateral spreading and vertical advance being determined by the soil hydraulic properties and emitter flux (Assouline, 2002; Skaggs et al., 2004). The importance of this geometry lies in the fact that it (i) determines the location of water-filled pore space (WFPS) large enough to limit oxygen diffusion, (ii) the root location where roots grow preferentially, and (iii) the duration of dissolved N and co-applied compounds in the active root zone. Hydraulic conductivity in coarse-textured soils may facilitate deeper percolation and reduce solute residence time of a given pulse volume, whereas finer-textured soils may keep water near the emitter and maintain greater areas of low gas diffusivity (Corona et al., 2026).

Such moisture gradients influence microbial activity by altering water-film connectivity, substrate diffusion, and oxygen transport in unsaturated soil. Water films, diffusion path lengths and oxygen supply in partially saturated pore networks can vary dramatically across centimeters, creating so-called patchy metabolic opportunities despite uniform bulk soil conditions (Or et al., 2007). In other words, a drip system does not simply alter the average moisture; it may change the spatial template within which microbial processes are carried out. Because ammonia oxidation is an aerobic process, nitrification generally occurs in oxygenated microsites; however, its rate depends jointly on oxygen availability, soil moisture, NH_4_^+^ supply, and microbial activity. Excessive wetness may restrict oxygen diffusion, whereas excessive dryness may limit substrate diffusion and microbial connectivity (Lacroix et al., 2021).

Emitter placement adds an additional layer of structure. Subsurface emitter placement may cause the wettest areas to be deeper, which can tend to decrease surface evaporation and reposition the oxygen-limiting core and the point at which roots meet the steepest moisture gradients (Schwartzman and Zur, 1986; Assouline, 2002). The overall result is that the design of drip systems is not a single treatment in itself, but a set of physical boundary conditions which shape the reactor volume within which N transformations occur.

### 3.2 Chemical gradients, nitrogen partitioning, and solute transport

The wetted bulb may organize solute transport by coupling short, localized high-intensity infiltration processes with massive concentration gradients. Drip irrigation experiments demonstrate that water application by emitters may lead to the creation of strong spatial patterns of solutes distribution and retention, through a combination of advection during irrigation and diffusion/redistribution afterward (Danierhan et al., 2013; Wang et al., 2019). For mineral N, this favors strong near-emitter gradients after fertigation, followed by redistribution according to the moisture gradients as well as root uptake zones. Since fertigation transfers N in a dissolved state, the high N concentrations may occur at the start of irrigation, yet their persistence depends on the volume of the irrigation pulse, soil water redistribution, and uptake demand between irrigation pulses (Cote et al., 2003).

Evidence indicates that the wetted bulb can spatially separate mineral-N forms, although the resulting pattern is strongly context dependent. In controlled experiments on NH_4_^+^-NO_3_^-^ fertigation, NH_4_^+^ remained concentrated close to the point source, whereas NO_3_^-^ was more broadly distributed and accumulated near the wetted boundary (Li et al., 2003). Notably, the wetted bulb tends to separate N pools in both space and time: NH_4_^+^ tends to remain near application areas due to cation exchange and slow movement as compared to NO_3_^-^, which may move either to the periphery of the bulb or to deeper layers when drainage occurs. The field evidence from subsurface drip irrigation demonstrates that irrigation management alters the spatial distribution of inorganic N and associated biogeochemical indicators, with the clearest differences occurring at specific soil depths rather than following a universal zone pattern (Schmidt et al., 2018). Thus, zones enriched in NH_4_^+^ and NO_3_^-^ may develop within the wetted bulb, but their persistence and location depend on soil properties, irrigation management, fertilizer form, and crop uptake. This context-dependent partitioning is relevant to nitrification control because ammonia oxidation depends on the temporal and spatial coincidence of NH_4_^+^ supply, suitable moisture, adequate oxygen, and active ammonia oxidizers (Ghasemi et al., 2023).

The second implication is methodological: bulk soil sampling can smooth over the chemically distinct microdomains and obscure the true location of nitrification which may help explain why the same rate of fertigation gives different nitrate profiles under different drip designs (Cote et al., 2003; Schmidt et al., 2018). As a practical minimum for surface drip systems, soil may be sampled along emitter transects at approximately 0, 10, 20, and 30 cm from the emitter, and at 1 m depth. Comparable schemes have been used to determine spatial water and mineral-N distributions under drip fertigation (Zain et al., 2023; Feng et al., 2022). For subsurface drip systems, the vertical sampling intervals should additionally consider the emitter burial depth, with samples collected above, adjacent to, and below the drip pipe. However, these distance from the emitter and depth should be adjusted to the observed wetting radius, emitter discharge, irrigation amount, and soil properties rather than applied as a universal fixed grid. Spatially resolved sampling (distance between emitter and depth) is therefore not a luxury in drip studies: it is often necessary to interpret N transformation results in a mechanistic way.

### 3.3 Microbial ecology: the wetted bulb as a predictable “hotspot” reactor

The wetted bulb naturally produces spatial hotspots (around emitters, roots, and interfaces) and temporal hotspots (following wetting pulses) which contribute to the overall system-level fluxes (McClain et al., 2003). Physically, drip concentrates water and substrates into a limited volume, while leaving adjacent soil relatively dry, thus intensifying gradients and concentrating microbial turnover where water, N and labile carbon co-occur (Or et al., 2007; Schmidt et al., 2018). These gradients provide a plausible basis for spatial and temporal hotspots associated with irrigation or fertilization events and emitter position. However, direct measurements of denitrification and nitrification hotspots within the wetted bulb remain limited, and their predictability should be considered a testable hypothesis. A framework of rhizosphere process control in drip-irrigated wetted bulb is presented in **Figure 2**.

**Figure 2:**
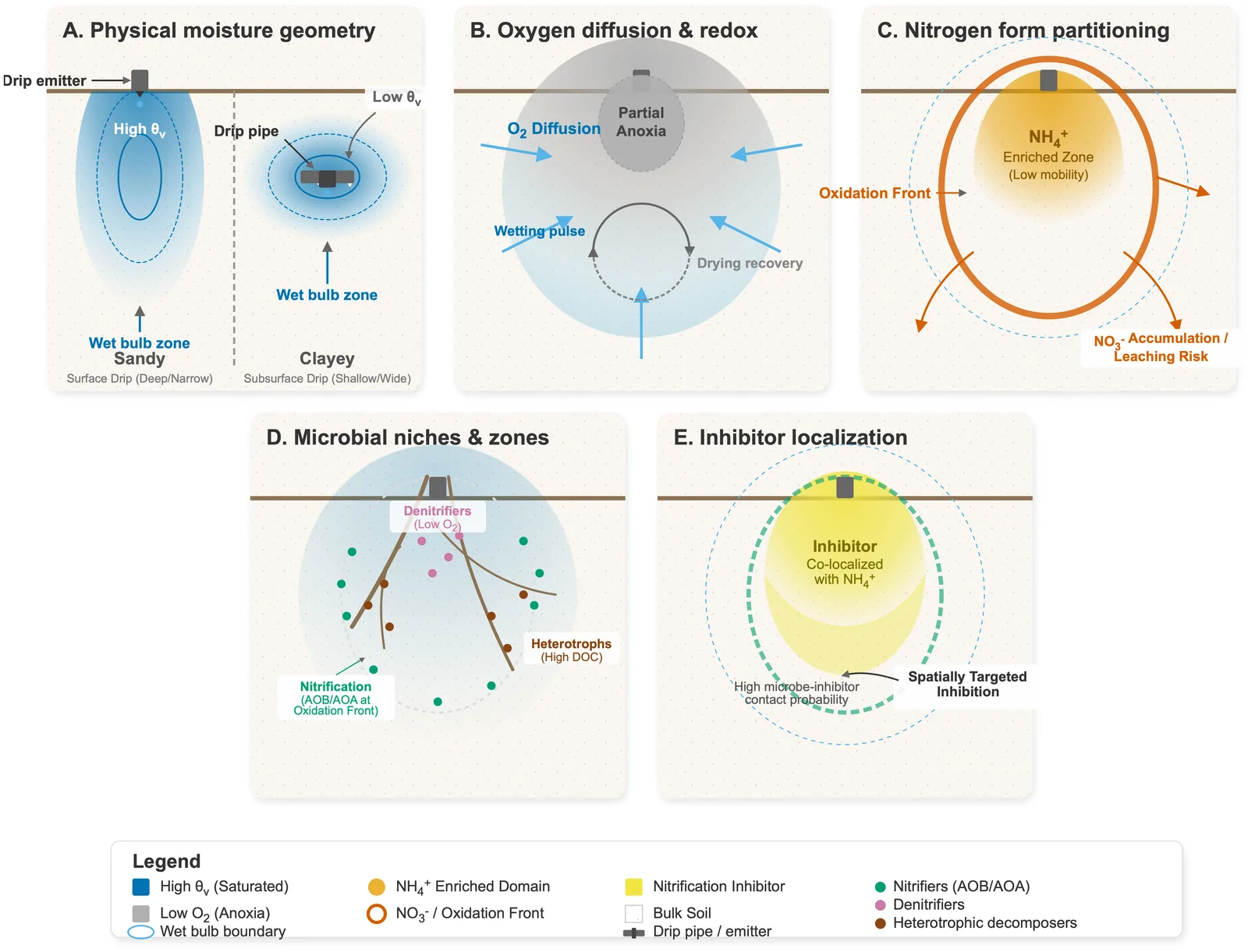
Conceptual framework of rhizosphere process control within the drip-irrigated wetted bulb. **(A) **Physical moisture geometry under contrasting wetted-bulb configurations and soil textures; **(B)** oxygen diffusion and redox dynamics during wetting and drying; **(C)** spatial partitioning of NH_4_^+^ and NO_3_^-^ and the associated oxidation front; **(D)** distribution of microbial niches within the wetted bulb; **(E)** inhibitor localization and co-localization with NH_4_^+^. The wetted bulb, defined as the localized, three-dimensional volume of soil wetted by the drip irrigation emitter (represented by grey rectangles) creates distinct physicochemical gradients that govern nitrogen transformations in the rhizosphere.

Indirect and system-level evidence is consistent with this hypothesis, although it does not confirm the persistence of microbial hotspots within the wetted bulb. Consistent with this mechanism, a global meta-analysis of field studies has reported that, on average, drip irrigation systems emit less N_2_O than furrow or sprinkler systems, though responses vary depending on climate, soil, and fertilizer regimes (Kuang et al., 2021). At the process scale, a drip-fertilized maize system demonstrated that the vertical placement of water and N (surface vs subsurface) can shift N_2_O emissions and N-cycling gene abundances, which means that irrigation-created habitat structure propagates into microbial functional potential (Guardia et al., 2024). The detailed spatiotemporal observations further support the idea that dynamics of N transformation under drip fertigation are not homogeneous, but they change over time as localized wetting, substrate delivery, and root uptake interact (Bi et al., 2022). Together, these studies support treating the wetted bulb as a semi-controlled biogeochemical reactor, whose reaction field can be shaped with the help of irrigation design and scheduling. Most importantly, the same spatial structuring may create narrow interfaces where the processes of nitrification and denitrification couple efficiently; however, spatially resolved measurements are required to confirm their management potential, location and persistence.

### 3.4 Implications for nitrogen loss mitigation

Once the wetted bulb is considered as the primary hotspot where N transformations occur, the placement becomes a mechanistic concept instead of a generic 4R slogan. The size and overlap of wetted volumes depend on emitter spacing and discharge (Schwartzman and Zur, 1986; Li et al., 2004a), whereas the irrigation frequency and duration determine whether the reactor favors aerobic nitrification, coupled nitrification-denitrification, or increased plant capture. Modeling frameworks (e.g., HYDRUS-based simulations) offer a method of mapping how management decisions translate into moisture and solute fields at the scale where the response of microbial processes occurs (Skaggs et al., 2004). Because these processes are three-dimensional in nature, drip experiments whose results report only total irrigation (without pulse structure, emitter depth, and emitter spacing) may not have the characteristics required to interpret the results of nitrification and N loss in any mechanistic way.

This reasoning directly motivates combining drip with NIs: inhibitors are most effective when they co-exist with active ammonia oxidizers in the microsites where NH_4_^+^ and oxygen coincide. Combining drip irrigation with a nitrification inhibitor has been reported to decrease N_2_O emissions in irrigated maize under production conditions similar to those of uninhibited fertilization, which confirms that the context of water delivery can precondition the performance of nitrification inhibitors (Guardia et al., 2017). A similar drip-fertilization context in perennial orchards indicates that the management of the irrigation regime and N form can also significantly influence the greenhouse gas production, reinforcing that moisture dynamics and N process control are inseparable in drip-irrigated soils (Maris et al., 2015). Mechanistically, drip can minimize losses by decreasing the reactive soil volume, whereas inhibitors can minimize losses by slowing the conversion of NH_4_^+^ to NO_3_^-^; the combined outcome is a function of whether transport (water fluxes) and transformation (microbial kinetics) occur in the same zones.

## 4 Nitrification inhibitors: mechanisms, fate, and context dependence

Nitrification inhibitors (NIs) are usually used to suppress microbial ammonia oxidation, thereby slowing the conversion of NH_4_^+^ to NO_3_^-^ and potentially minimizing the amount of nitrate leaching and denitrification, while prolonging the NH_4_^+^ availability for root uptake (Abalos et al., 2014; Ruser and Schulz, 2015). This is a compelling logic, but field outcomes vary because nitrification is not a bulk-soil phenomenon, and NIs effectiveness depends on maintaining an effective concentration at active ammonia oxidation sites (Beeckman et al., 2024). This coincidence problem is more designable in drip-fertigated soils, but also more conditional: the wetted-bulb gradients outlined in Section 3 can stabilize an oxic-NH_4_^+^ interface, but only if inhibitor fate, irrigation waveform (defined here as the temporal pattern of water delivery determined by irrigation-event frequency, duration, emitter discharge and water volume or depth applied per event) and fertigation placement keep NI exposure aligned with that oxic-NH_4_^+^ interface.

### 4.1 Biological targets and pathway-level specificity of inhibitors

The most commonly used synthetic NIs (such as dicyandiamide (DCD), DMPP, nitrapyrin, and 3,4-dimethylpyrazole succinic acid (DMPSA)) share the functional outcome of suppressing ammonia oxidation but differ in their chemical properties, microbial selectivity, soil fate, and biochemical modes of action (Chaves et al., 2006; Benckiser et al., 2013). Mechanistically, this is important since ammonia oxidation is the gateway that connects the fertilizer-derived NH_4_^+^ to the mobile and loss-prone nitrate pool. If ammonia oxidation is retarded while roots are active, the same N input can be diverted from “rapid conversion and transport” toward “retention and uptake”, which is the central agronomic promise of NI use. In the dimethylpyrazole-based inhibitors (DMPP/DMPSA), evidence supports a mechanism linked to the micronutrient chemistry: these compounds (or their active ligand forms) can complex copper, a cofactor essential for ammonia oxidation, and this offers a mechanistic bridge between the inhibitor action and the availability of trace metals in the soil (Corrochano-Monsalve et al., 2021). This proposed mechanism is important in the sense that it reframes the NIs “mode of action” from a generic narrative of toxicity as a contextual biochemical interaction that may be different in different soils depending on soil chemistry. However, pure-culture evidence also indicates that 3,4-Dimethyl-1 H-pyrazole (DMP) can directly and reversibly inhibit ammonia monooxygenase, while soil studies show that ammonia-oxidizing bacteria, archaea, and comammox may differ in their sensitivity to DMPP (Kleineidam et al., 2011; Yildirim et al., 2023). Thus, the mode of action is likely to involve interacting biochemical and microbial mechanisms whose relative importance varies with soil chemistry and nitrifier community composition.

Many studies report stronger inhibition of ammonia-oxidizing bacteria (AOB) than ammonia-oxidizing archaea (AOA), but this pattern is not universal, and the responses of comammox Nitrospira may also vary with soil conditions, inhibitor type, and nitrifier community composition (Tao et al., 2021a; Yin et al., 2023; Chai et al., 2025). Instead of considering this a fixed taxonomic response, a process-control frame considers it a stability issue: because the dominant ammonia oxidizers shift across moisture and substrate niches within a season, the same nitrification inhibitor can produce different nitrification responses without any change in dose. A global meta-analysis of functional genes indicated significant decreases in AOB ammonia monooxygenase subunit A gene (amoA) abundance with the application of NIs, whereas AOA amoA responses were smaller or not significant in many cases; however, changes in functional-gene abundance do not necessarily indicate equivalent changes in process contribution or microbial activity (Yin et al., 2023). This specificity is important in drip systems as the wetted-bulb gradients may shift the relative significance of the AOB versus AOA niches across oxygen and moisture microzones. The supply of NH_4_^+^ can be high near the emitter and oxygen diffusion may be limited intermittently after irrigation; conversely, near the bulb margin, oxygen recovers faster but NH_4_^+^ supply may be attenuated (Zhou et al., 2021; Qiao et al., 2023). The effective target zone of inhibition could therefore be the moving overlap of NH_4_^+^ and oxygen, not the entire wetted volume. Practically, it implies that a single dose of nitrification inhibitor can yield varying nitrification results at varying distances from the emitter or at varying intervals between irrigation pulses even in the same field.

### 4.2 Sorption, mobility, degradation kinetics under dynamic soil moisture

NI performance is limited as much by fate as potency. After NI application, inhibitors partition among the soil solution, mineral and organic surfaces, and microbial biomass, while simultaneously undergoing abiotic transformation and biodegradation (Ruser and Schulz, 2015; Bozal-Leorri et al., 2022). Their effectiveness in the nitrification zone therefore depends on transport and persistence under the local water regime. This is of particular importance under drip irrigation because the moisture distribution is non-uniform: the wetting fronts, drainage and localized evaporation create time-varying gradients that regulate both inhibitor movement and microbial activity.

A key implication in drip contexts is that “persistence” is spatially structured. The inhibitor may persist in bulk soil for a substantial period after application, yet remain functionally ineffective if it is diluted at the oxidation front, transported beyond the active root zone, or retained in zones where ammonia oxidation is already oxygen-limiting. In other words, agronomically relevant persistence is residence time in the nitrification-active microzone rather than persistence in the profile. Differences among inhibitors are practically important. DCD is typically more mobile in the soil solution than the dimethylpyrazole-based compounds, making it more susceptible to displacement under preferential flow or strong percolation (Marsden et al., 2016). By contrast, sorption and chemical interactions, such as copper-related chemistry, tend to be stronger influences in DMPP/DMPSA behavior (Corrochano-Monsalve et al., 2021; Bozal-Leorri et al., 2022). In a controlled soil incubation, DMP supplied as DMPSA reached its maximum concentration approximately 8 days after application, whereas DMP supplied as DMPP almost disappeared within 30 days, demonstrating the delayed activation of DMPSA (Bozal-Leorri et al., 2022).

Temperature and water status further modulate persistence, as moisture and temperature can have a significant impact on the duration of nitrification inhibition through their effects on degradation kinetics and biological turnover (Menéndez et al., 2012). For example, the estimated half-life of DCD decreased from 89 days at 5 °C to 37 days at 15 °C and 18 days at 25 °C across nine UK soils (McGeough et al., 2016). In another study conducted at 15 °C, DCD and DMPP half-lives ranged from 20–33 and 12–17 days, respectively, depending on organic matter and soil clay content (Chibuike et al., 2022). Under drip irrigation, irrigation scheduling further complicates these dynamics by creating repetitive wetting-drainage cycles that can periodically facilitate inhibitor transport (during pulses) and concentrate solutes during redistribution and evaporation. These processes can alter the history of inhibitor exposure by nitrifiers, even within a given week.

### 4.3 Biophysical mechanisms of inhibitor failure

Major failure modes include dilution through over-irrigation, displacement by bypass flow as along macropores, or movement outside the primary nitrification zone, especially for more mobile compounds (Ruser and Schulz, 2015; Marsden et al., 2016). A second failure mode is temporal mismatch: NI application is most beneficial within the window of co-occurrence between NH_4_^+^ and oxygen and when the ammonia oxidizers are active, yet most management strategies implement NIs without aligning with the temporal dynamics of wetting, drainage, and root uptake that constitute that window (**Figure 3**). Drip systems may lower this mismatch through co-delivery NIs with fertigation pulses, but may also increase this mismatch in cases where pulse design generates short-lived, shifting oxidation fronts which are not captured by a single event of inhibitor placement.

**Figure 3:**
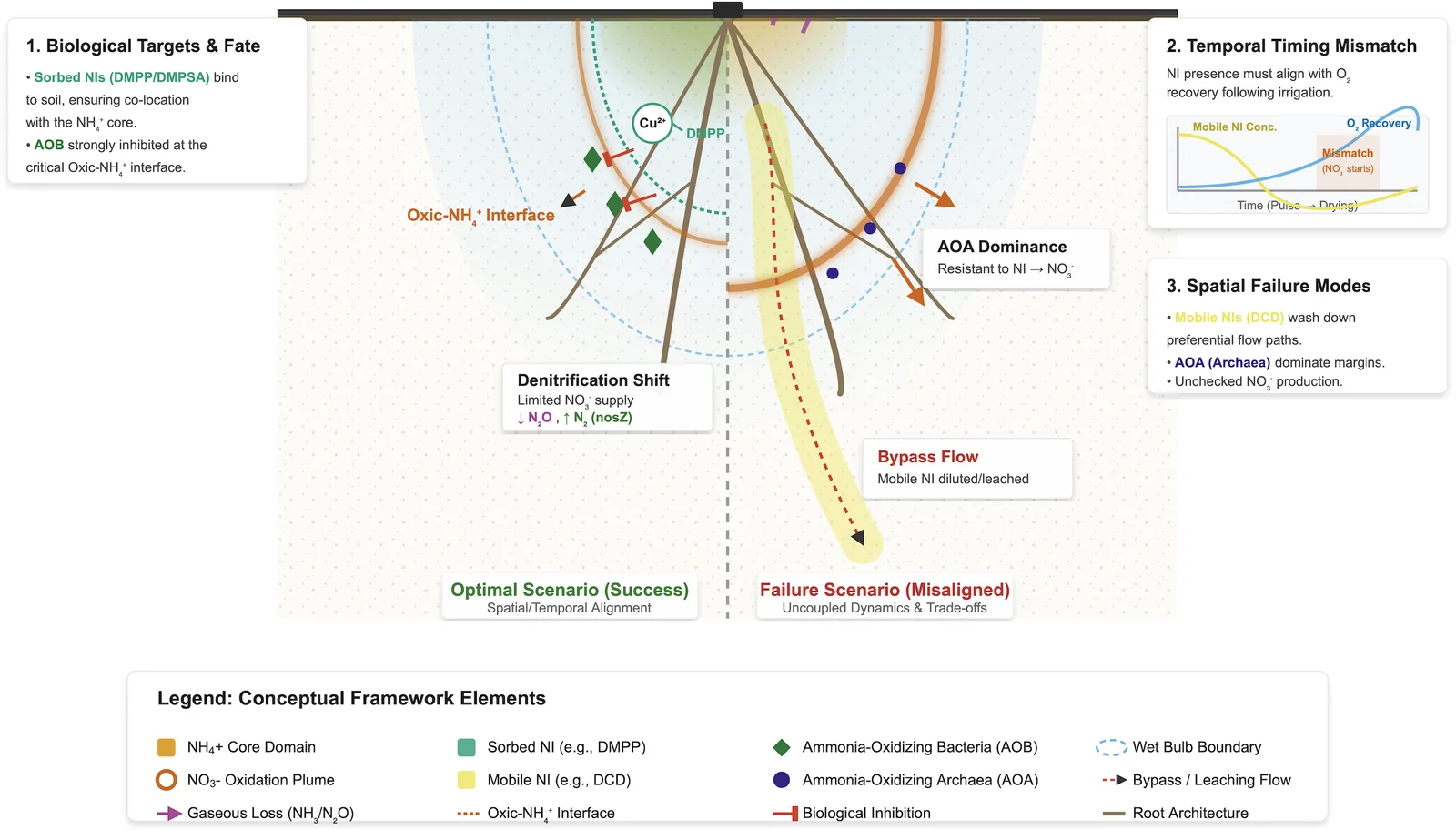
Conceptual schematic illustrating the context-dependent efficacy of NIs under drip fertigation. The diagram contrasts an optimally aligned scenario (left) with a misaligned failure scenario (right) within the spatial gradients of a drip-irrigated wetted bulb.

The third limitation is process substitution and substrate limitation. When nitrification is already low (for example, under low oxygen levels in persistently wet microsites), further inhibition might also be ineffective as nitrification is no longer the limiting step due to low oxygen (Tan et al., 2018; Cao et al., 2025). Finally, repeated or longer-term use increases the possibility of community changes that alter the apparent inhibitor efficacy (such as changes in nitrifier community structure or relative proportions of AOA and AOB), although strong, generalizable evidence of real adaptation has yet to be found (Ruser and Schulz, 2015; Yin et al., 2023).

### 4.4 Co-process interactions and biogeochemical feedbacks

Since NIs increase the residence time of NH_4_^+^, they can create trade-offs. Ammonia (NH_3_) volatilization is one risk that has been widely discussed, especially in high-pH soils or where fertilizer application causes NH_4_^+^ to be in close proximity with the atmosphere (Lan et al., 2022). A global meta-analysis evaluating NH_3_ volatilization showed that NIs may enhance NH_3_ losses in most environments and the magnitude of this effect is highly dependent on soil pH, the type of inhibitor, fertilizer, and experimental conditions (Wu et al., 2021). This matters under drip management because subsurface placement may reduce NH_3_ exposure to the atmosphere, but fertilizer chemistry (including localized pH and carbonate buffering) remains the determinant of NH_4_^+^ presence as NH_3_ in the soil solution at gas exchange interfaces. A similar reduction in NH_3_ volatilization was reported following subsurface placement of controlled-release blended fertilizers in rice fields, although the study did not evaluate drip fertigation (Ahmad et al., 2025).

NIs also affect denitrification indirectly through their effects on the NO_3_^-^ supply. They can slow down nitrate formation, resulting in limited availability of denitrification substrates, and thus decreased emissions of N_2_O in nitrate-limited microsites (Wu et al., 2017). However, the outcome is not merely less denitrification. In some soils, NIs may also alter the product ratio by shifting redox and nitrate conditions in soils that influence N_2_O reduction. For example, the work linking nitrification inhibitor application to changes in the dynamics of the nitrous oxide reductase gene (nosZ) and N_2_O:N_2_ partitioning supports the idea that part of the mitigation effect can be due to a shift toward more complete denitrification (more N_2_, less N_2_O) rather than only reducing total denitrification (Friedl et al., 2020). In spatially structured wetted bulbs, this coupling is naturally zonal: oxygen-rich regions determine where nitrification and its inhibition are most important, while wetter, oxygen-limiting regions determine whether any NO_3_^-^ formed is reduced to N_2_O or further reduced to N_2_.

Taken together, NI performance depends on the dominant ammonia oxidizers, inhibitor persistence, and redistribution among downstream N-loss pathways. These dependencies provide the mechanistic basis for the integrated framework developed in the following section.

## 5 Integrating drip irrigation and nitrification inhibitors: from input application to designed biogeochemical function

Drip irrigation was developed to enhance delivery: place water and dissolved nutrients more frequently and efficiently close to roots. But drip systems do not only deliver inputs. They impose repeatable gradients in moisture, oxygen, and nitrogen forms by means of concentrating water and solutes into a bounded wetted bulb, where nitrification is most likely to occur (Kuang et al., 2021; Guardia et al., 2024). This creates an opportunity to shift away from “precision input” to “precision process control” in which management aims to regulate the soil reactions that convert N fertilizer into mobile nitrate and gaseous losses.

Nitrification inhibitors target the gatekeeping step of aerobic N transformation by inhibiting ammonia oxidation. Yet NI performance varies across soils (Shi et al., 2017; Zhu et al., 2019), moisture conditions (Hu et al., 2015; Fan et al., 2019), and fertilizers (Pereira et al., 2010). The central argument advanced here is conceptual rather than directly demonstrated; drip irrigation may alter the geometry of inhibitor effectiveness by potentially increasing the spatial and temporal overlap among (i) the presence of inhibitor, (ii) the supply of NH_4_^+^, and (iii) active ammonia oxidizers, thereby transforming NIs from diffuse field inputs into a targeted regulators within a defined reaction domain. This interpretation is mechanistically plausible and consistent with observed effects of moisture dynamics and drip configuration, but direct, spatially resolved measurements of simultaneous overlap among these components within the wetted bulb remain limited. A useful way to frame this coupling is to consider the wetted bulb as reactor with a characteristic “oxidation front”. The bulb core usually has the wettest zone after irrigation which may be relatively oxygen-limited, whereas the bulb margin and draining areas after irrigation are likely to become the sites of co-occurring oxygen and NH_4_^+^ where ammonia oxidation accelerates. Within this framing, the goal behind drip-NIs integration is not merely to “apply inhibitor with fertilizer” but to maintain inhibitory exposure at those locations and times when ammonia oxidation is most active inside the bulb. However, this spatial concentration is not inherently beneficial, poorly matched irrigation and NIs management may intensify oxygen limitation and salinity accumulation or increase the risks of ammonium toxicity and off-target inhibitor effects.

### 5.1 Drip fertigation may promote co-localization of NIs and NH_4_^+^ within the same reactive volume, potentially increasing contact with nitrifiers

Inhibition is not only controlled by the applied dose; rather, it depends on whether inhibitory concentrations coincide with active nitrification microsites. Under drip fertigation system, co-applied dissolved fertilizer N and NIs enter the same wetted soil volume, which may increase the likelihood that the inhibitor molecules will come into contact with ammonia oxidizers where substrate supply is greatest. However, their spatial distribution may diverge because of differences in sorption, mobility, and degradation. Spatial coincidence may be more difficult to achieve with broadcast application because a large fraction of the nitrification inhibitor mass may occupy soil volume with low ammonia availability or low nitrifier activity.

This proposed co-localization mechanism is indirectly supported by field studies, although it has not yet been directly demonstrated. Application of DMPP decreased N_2_O emissions and temporarily lowered the abundance of N-cycling genes in fertigated maize during the fertigation period; furthermore, contrasts between surface and subsurface drip reflect how irrigation depth changes the mineral N distribution and microbial habitat under drip systems (Guardia et al., 2024). In controlled experiments that directly compared surface and subsurface irrigation across diverse soil textures, inhibitor behaviors varied with wetting pattern and texture, which supports the idea that the interactions between delivery platform and inhibitor chemistry can produce significant changes in nitrification control and trace-gas results (Ribeiro et al., 2024). In another study on cotton, the application of nitrapyrin in plastic-mulched drip fertigation enhanced the availability of soil NH_4_^+^ and decreased seasonal N_2_O emissions and increased NUE, consistent with enhanced suppression of ammonia oxidation in the wet area (Liu et al., 2017). Thus, available evidence suggests that the effectiveness of NIs may vary with drip configuration and soil conditions, but they do not establish spatial coupling as the causal mechanism or confirm that the effect is consistent across soils, crops, and inhibitor formulations.

### 5.2 Pulsed irrigation-based hydro-redox cycling and its potential effects on NI persistence and efficacy

Hydro-redox cycling refers to irrigation-driven changes in soil moisture content that alter oxygen diffusion, air-filled porosity, and redox conditions during wetting and subsequent drainage. Drip irrigation introduces a management lever that the broadcast systems lack: the irrigation waveform (frequency × duration × depth) defines the dynamics of short-term oxygen diffusion and recovery within the wetted bulb. When an irrigation pulse increases WFPS, oxygen diffusion may be restricted and nitrification may temporarily decline. As soil drainage proceeds, oxygen supply may recover, allowing nitrification to increase again. However, not every drip-irrigation event produces a pronounced redox cycle; its magnitude and duration depend on irrigation depth and antecedent soil water content, soil texture, and biological oxygen demand. The practical implication is that inhibitors may be most beneficial when they persist into such post-irrigation “reactivation windows”, when ammonia oxidizers would otherwise increase substrate turnover, provided that sufficient NH_4_^+^ remains available and inhibitor concentrations remain effective.

Mechanistic work demonstrates that in addition to inhibition of nitrification, moisture conditions can also alter subsequent downstream N_2_O dynamics, including shifts in how nitrification inhibition interacts with processes of denitrification as well as N_2_O reduction capacity (Torralbo et al., 2017). However, that study compared contrasting moisture conditions rather than repeated irrigation-driven wetting and drainage. A greenhouse drying-rewetting experiment further showed that NI responses were soil-dependent; DMPSA and DMPP reduced total N_2_O emissions by 90% and 88%, respectively, in a calcareous soil, whereas nitrification activity was low in the acidic soil (Sánchez-Martin et al., 2024). This indicates that wetting-drying history alone does not ensure effective inhibition.

Evidence obtained from drip-managed fields is consistent with the idea that irrigation scheduling and inhibitor use are coupled controls rather than independent interventions (Li et al., 2026). Under film-mulched drip fertigation in an arid region, the combination of urease and nitrification inhibitors affected soil N retention and reduced greenhouse gas production compared with uninhibited practice, with outcomes associated with irrigation management and the cyclic nature of fertigation (Sunling et al., 2024). However, because film mulch, drip fertigation, and both urease and NIs were combined, the specific contribution of drip-NIs integration cannot be isolated. These findings are consistent with, but do not directly demonstrate, a process-control explanation: irrigation pulses influence the oxygen and transport context, while inhibitor chemistry may influence the strength with which the system suppresses ammonia oxidation during the periods when oxidation would otherwise occur. The effects of NI application under drip irrigation systems in different crops are summarized in **Table 2**.

**Table 2:** Comprehensive synthesis of field and greenhouse studies evaluating the agronomic, and environmental efficacy of NIs co-applied via drip irrigation in different crops.

Crop	Nitrification inhibitor and co-applied treatment (s)	Drip configuration and experimental setting	Key results	Limitations of the study	Research recommendations	Citation
Olive orchard	DMPP	Surface and sub-surface drip irrigation, field experiment	• DMPP reduced cumulative N_2_O. • Subsurface drip system mitigated N_2_O flux than surface drip irrigation.	*	The studies covering more sampling intensity are required.	Maris et al., 2015
Maize	DMPP	Surface and sub-surface drip irrigation, field experiment	• DMPP influenced nitrifying and denitrifying genes. • DMPP was the most effective strategy to abate N_2_O fluxes.	*	Relationships of N_2_O with nitrifiers, denitrifiers, and mineral N should be evaluated across soil profile.	Guardia et al., 2024
Maize	DMPP	Surface and sub-surface drip irrigation, field experiment	• Sub-surface drip and nitrification inhibitor increased maize grain yields by 12 % as compared to surface drip, also increases grain N content.	Crop yield and phenology was not observed during transition year from rainfed to irrigation management.	Need to better understand the maize yield and N uptake by measuring N loss pathways.	Monistrol et al., 2024
Maize	DCD; Nitrapyrin	Surface drip irrigation, field experiment	• NI treatments under drip fertigation reduced annual cumulative N_2_O by up to 66% and NO by 95% than flood irrigation without NI.	*	*	Tian et al., 2017
Wheat	DCD; DMPP; MP + TZ	Surface and sub-surface drip irrigation, pot experiment	• Sub-surface drip reduced total N_2_O by 44-52% than surface drip; DMPP most effective in N_2_O mitigation.	*	Results should be further confirmed under various field conditions.	Ribeiro et al., 2024
Maize	DMPSA; NBPT	Surface drip irrigation, field experiment	• NBPT decreased N_2_O loss by 42% than without inhibitor and drip fertigation. • DMPSA reduced 60% N_2_O loss than conventional planting (without inhibitor and drip fertigation).	*	*	Sunling et al., 2024
Cotton	Nitrapyrin	Surface drip irrigation, field experiment	• Urea plus nitrapyrin under drip fertigation reduced seasonal N_2_O emission by 32.4% and improved NUE by 10.7% as opposed to urea alone.	*	Future work is required to evaluate whether denitrification dominates nitrification under pulse drip fertigation.	Liu et al., 2017
Citrus	DMPP	Surface drip irrigation, field experiment	• Ammonium sulfate plus DMPP improved N fertilization efficiency and reduced leaching risk as compared to the alone ammonium sulfate application.	*	*	Quinones et al., 2009
Citrus	DMPP	Surface drip irrigation, lysimeter experiment	• Ammonium sulfate and DMPP achieved highest NUE (69%) than 54% NUE in alone ammonium sulfate. • Less than 3% of applied fertilizer was leached under DMPP as compared to 9-10% losses under ammonium sulfate application.	*	In future studies, DMPP application should be considered in spring season.	Martínez-Alcántara et al., 2013
Winter wheat	DMPP	Surface drip irrigation, lysimeter experiment	• DMPP reduced N_2_O emissions by 15.05%, 18.96%, and 11.27% under high, medium and low irrigation respectively. • DMPP caused suppression of AOB amoA gene by 22.41%, 47.2%, and 29.73% under different soil moisture regimes.	*	Studying community structure of microbes could provide additional insights.	Liang et al., 2024
Maize	DMPP (with alternate drip irrigation)	Sub-surface drip irrigation, rain shelter field experiment	• DMPP plus alternate drip irrigation reduced N_2_O flux by 37.4-70.4% and increased NH_3_ volatilization by 13.5-18.7% as compared to no DMPP application.	*	Long term ecosystem responses and functional nitrogen cycling genes should be examined.	Li et al., 2026
Maize	DMPSA	Surface drip irrigation, field experiment	• Drip-fertigation + DMPSA evaluated for N-oxide mitigation without yield penalty.	*	Urea substitution by CAN and urease inhibitors must be evaluated for N loss mitigations without affecting yield.	Guardia et al., 2017
Tomato-Melon rotation	DMPP; DCD (with/without microbial agent)	Surface drip irrigation, greenhouse experiment	• DMPP + microbial agent improved yield by 21.7% in melon and 41.5% in tomato as compared to local fertilization. • DMPP also improved tomato fruit quality (e.g., vitamin C + 65.5%).	Absence of fruit quality and microbiome network data limits interpretation of findings.	Further experiments required to verify the findings.	Pu et al., 2025
Cotton	DCD, and NBPT	Surface drip irrigation, field experiment	• Generally, no treatment effect on nitrogen allocations, cotton fiber quality, seed and lint yield.	Study conducted in dryland region.	Further need to verify whether the benefits of EENFs could be achieved under low N application rates.	Li et al., 2020
Wheat	Nitrapyrin; Nitrapyrin + NBPT (double inhibitors)	Surface drip irrigation, field experiment	• 20% reduced N + double inhibitors maintained the yield. • Highest reported NUE (43.6%) with application of double inhibitors.	*	Potential of combine use of nitrapyirn and NBPT should be examined against N losses in calcareous soils.	Tao et al., 2021b
Tomato	DMPP; polymer-coated urea	Surface drip irrigation, greenhouse experiment	• DMPP first improved tomato growth and yield, then decreased with increase in nitrogen application. • The WUE was also high as compared to urea-only treatment.	*	Research in wider area is required to evaluate the nitrogen application strategy.	Nie et al., 2022
Wheat	DMPP	Sub-surface drip irrigation, field experiment	• Balancing NH_4_^+^ and NO_3_^-^ supply through CAN + Urea + DMPP under drip fertigation improve overall agronomic efficiency of wheat.	DMPP was used only with NH_4_^+^ dominant fertilizer.	Further validation of the study under contrasting soil conditions.	Hao et al., 2026
Cotton	DCD, Urease inhibitor	Surface drip irrigation, field experiment	• SuperU (containing DCD and Urease inhibitor) application notably reduced CH_4_ uptake rate by 49.4% as compared to urea fertigation.	Concentration gradient method to determine CH_4_ uptake has limitations in drip system	Future studies should focus on refining calculation methods and monitoring equipment to assess CH_4_ diffusion.	Li et al., 2025
Blueberry	DMPP	Surface drip irrigation, pot experiment	• pH values of leachate were high in DMPP treatment as compared to without-DMPP treatment. • Addition of DMPP resulted in decreased biomass production than no DMPP application.	The mechanism causing low biomass production is not clear enough.	More comprehensive work on negative effects of high NH_4_ concentration should be carried out.	Tamir et al., 2025
Rice	DMPP and NBPT	Surface drip irrigation, field experiment	• NH_4_^+^ with DMPP increased nitrogen metabolic enzymatic activity than urea and NO_3_^-^ application. • NH_4_^+^ with DMPP increased the rice yield by 2.04-6.25% as compared to Urea application.	The NH_4_^+^ and Urea treatments were supplemented with DMPP and NBPT. No nitrification inhibitor or urease inhibitor was applied under NO_3_^-^ treatments.	Future research should focus on investigating how NH_4_^+^/NO_3_^-^ ratios affect canopy architecture and light interception in rice.	Song et al., 2026

DCD, Dicyandiamide; DMPP; 3,4-dimethylpyrazole phosphate; DMPSA; 3,4-dimethylpyrazole succinic. MP + TZ, 3-methylpyrazol combined with Triazol; NBPT, N-(n-butyl) thiophosphoric triamide; CAN, Calcium ammonium nitrate; EENFs, Enhanced efficiency N fertilizers sources. * Indicates that the information was not explicitly reported in literature by the authors.

### 5.3 Precision hotspot targeting through drip is a mechanistic shift, not a marginal gain

Broadcast nitrification inhibitor strategies implicitly assume a broad distribution of nitrification and that uniform inhibitor presence is the most effective route of control. Drip systems create a spatially structured reaction domain: nitrification tends to concentrate where the overlap of NH_4_^+^ and oxygen occurs within the wetted bulb and along its oxic margins. In this context, precision is not merely closer placement. It is the transformation of an inhibitor from a field-wide blanket to a treatment covering a physically defined habitat.

Available evidence indicates that irrigation method can influence the results of nitrogen loss. A study conducted in a plastic-film mulched, high frequency drip-fertigated cotton field found that drip irrigation with nitrapyrin had significantly lower (32.4%) seasonal N_2_O emissions and increased NUE (10.7%) as compared with urea application alone (Liu et al., 2017). These results were obtained under specific crop, soil, fertilizer and irrigation conditions and should not be interpreted as universally applicable effects. Field experiments comparing drip irrigation levels under DMPP application indicate that the wetting depth can change mineral N distributions and microbial indicators across soil layers, which gives a mechanistic explanation for why the performance of inhibitors and the location of nitrification cannot be inferred to be the same across irrigation architectures (Liang et al., 2024). Texture-sensitive responses under surface and subsurface drip systems show that hotspot location and persistence are not fixed background conditions but an emergent characteristic of wetting geometry and soil structure (Ribeiro et al., 2024). Taken together, these studies show variability across soil texture, wetting depth, and experimental context rather than a universal benefit of precision hotspot targeting.

Accordingly, added benefits are expected only when nitrification occurs in a predictable zone and inhibitor exposure persists through reoxygenation; prolonged oxygen limitation or deep percolation may eliminate this advantage.

### 5.4 Drip-NIs-based rhizosphere engineering for nitrogen loss mitigation

In order to make drip-NIs integration a specified process control, we further propose the DRM to link management knobs with measurable wetted-bulb conditions, rates of related microbial processes, and agronomic/environmental outcomes (**Figure 4**).

**Figure 4:**
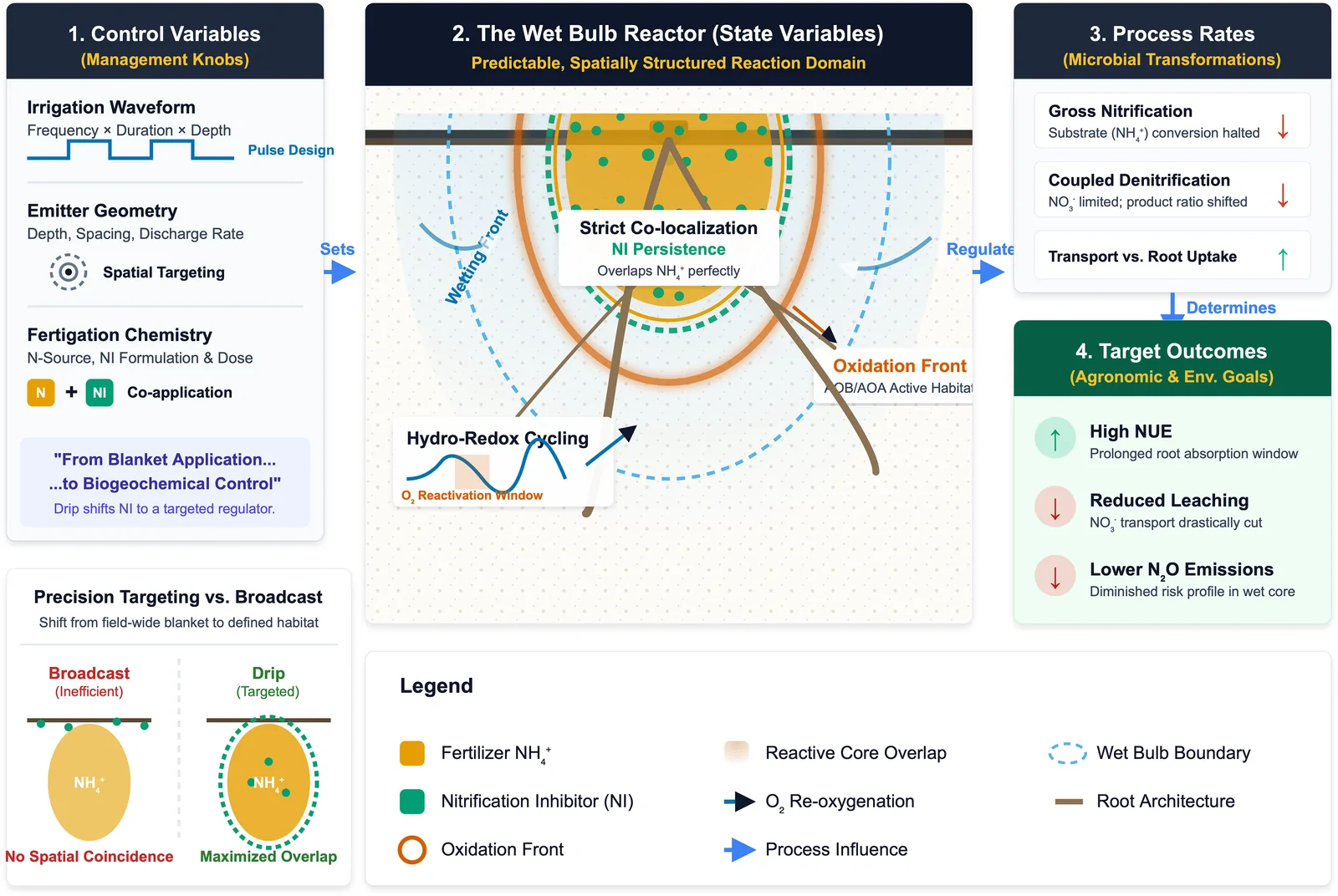
Conceptual Designer Rhizosphere Model (DRM) for drip-NIs integration. The framework illustrates the mechanistic pathway from agricultural inputs to targeted biogeochemical process control.

The control variables (design choices) include irrigation waveform; emitter depth, spacing, and discharge rate; fertigation chemistry and timing; and NI selection, formulation, and dosage strategy. These control variables set state variables within the wetted bulb, such as water content and oxygen availability, the partitioning of the NH_4_^+^/NO_3_^-^ fields, the concentration of inhibitors and their persistence, and the location of the nitrification-active oxidation front. These state variables regulate the process rates (gross nitrification, coupled denitrification and N_2_O reduction, and transport versus uptake), which then determine the subsequent outcomes, including NUE, the risk of nitrate leaching, and N_2_O emissions.

In practice, the DRM can be applied through a monitor-evaluate-adjust strategy. Measurable indicators may include crop nitrogen status, soil water content, NH_4_^+^ and NO_3_^-^ distribution, yield, and NUE, while research applications may additionally measure inhibitor residues, soil oxygen, nitrification activity, N_2_O emissions, and nitrate movement below the root zone. Key decision points include rapid NO_3_^-^ accumulation despite NI application, or prolonged NH_4_^+^ retention without corresponding crop uptake, increasing salinity, persistent oxygen limitation or inhibitor residues outside the target zone. Depending on the observed response, control variables can be adjusted according to crop demand, soil texture, and local climatic conditions.

The DRM provides a conceptual basis for developing hypotheses that can be evaluated through explicit field experiments. First, drip delivery may increase inhibition efficiency at an equal inhibitor mass by increasing the overlap between inhibitor presence and active zones of nitrification (as was observed under drip-configuration effects in maize and N-source-dependent responses under surface versus subsurface drip irrigation) (Monistrol et al., 2024). Second, irrigation waveform may regulate inhibitor performance by controlling oxygen recovery and inhibitor exposure dynamics (supported by drying-rewetting sensitivity and moisture-dependent inhibitor effects) (Torralbo et al., 2017; Sánchez-Martin et al., 2024). Third, texture may influence the optimal inhibitor strategy through variation in wetted-bulb geometry, drainage, and oxygen supply, as suggested by differences in inhibitor effects in contrasting soil textures under drip systems (Ribeiro et al., 2024). The cited studies support the effects of drip configuration, soil texture, fertigation timing, and moisture regime on NI performance, but they do not directly verify spatial co-location among NH_4_^+^, NIs, and active nitrifiers.

Finally, cyclic fertigation processes may result in better inhibitor performance when dosing is synchronized with the fertigation input and the oxidation front is kept within the active root zone, as evidenced in film-mulched drip fertigation under arid conditions (Sunling et al., 2024).

Crop characteristics may also result in variations in the effectiveness of drip-NIs systems. For example, photosynthetic response, root development, yield, and nitrogen efficiency of greenhouse-grown tomato varied with crop growth stage and nitrogen rate (Nie et al., 2022). Similarly, reducing nitrogen input while applying nitrapyrin with NBPT maintained wheat crop productivity and improved nitrogen use efficiency under drip irrigation, indicating that inhibitor benefits depend on stabilized nitrogen availability in line with seasonal crop demand (Tao et al., 2021b). Further, crop-specific responses were also evident in wheat and maize under drip-NIs fertigation systems, where the two crops differed in their yield responses to inhibitor treatments (Tian et al., 2017). Evidence from a rice-wheat rotation also showed that reduce N rated maintained grain yield in most crop seasons, although wheat was more sensitive to N reduction than rice, further emphasizing the need to align N management with crop-specific demand (Nadeem et al., 2022). Therefore, crop root distribution, nitrogen demand, phenology, and other management practices should be treated as explicit modifiers when applying the DRM across production systems. Beyond nitrification control, these interactions also influence the nitrogen distribution among crop uptake, volatilization, leaching, and gaseous-loss pathways, as discussed in the following section.

## 6 Whole-system consequences beyond nitrification

### 6.1 Synchronized fertigation restricts nitrate leaching, NH_3_ volatilization, and gaseous N losses

The strongest “whole-system” case for drip-NIs integration is not just slower nitrification, but a change in the distribution of reactive nitrogen among leaching, volatilization and gaseous pathways once fertilizer gets into the root zone. Drip fertigation itself can cause reductions in the fraction of soil exposed to high mineral-N concentrations by confining inputs to the wetted bulb. A large meta-analysis across the Chinese agricultural systems (443 observations) found substantial decreases in nitrate leaching and N_2_O emissions alongside an increase in yield and a small increase in nitric oxide (NO) emissions, which indicates that localized wetting can redistribute losses rather than uniformly reducing all gaseous products (Zheng et al., 2023). On a broader scale, a global meta-analysis shows that drip irrigation produces lower N_2_O emissions than sprinkler and furrow irrigation, which is in line with low-frequency large anoxic events and small reactive soil volumes under localized wetting (Kuang et al., 2021).

Adding nitrification inhibitors overlays a process control with this transport control: by slowing down the oxidation of NH_4_^+^, NIs decrease the rate at which mobile nitrate is produced within the bulb, narrowing the time frame in which nitrate fronts form and migrate. Across various farming systems, a meta-analysis indicates that nitrification and urease inhibitors can reduce reactive N losses and tend to increase NUE, yet responses depend on moisture, temperature, and management context (Abalos et al., 2014). However, these meta-analyses evaluated drip irrigation and NIs as separate interventions; they therefore support the possibility of combined management. In drip systems, particularly the key whole-system question is not only whether N_2_O emissions decrease, but whether (i) the progression of nitrate fronts is slowed, (ii) the risk of NH_3_ volatilization is kept low due to subsurface delivery and solution chemistry, and (iii) NO and N_2_O respond in the same direction, or exhibit trade-offs. The practical implication for the DRM is that the mitigation claims should be reported as a comprehensive set of pathways (NO_3_^-^ leaching, NH_3_ volatilization, N_2_O and NO) because a reduction in one of the pathways can trigger a compensatory increase in another pathway (Butterbach-Bahl et al., 2013). The minimum set of field measurements should therefore include: mineral-N profiles (NH_4_^+^ and NO_3_^-^) resolved through the bulb, at least one direct gaseous flux measurement (N_2_O, preferably with NO), and a proxy of leaching (drainage concentration or lysimeter-based loss), all of which should be reported on an area- and yield-scaled basis.

### 6.2 Sustained ammonium enhances productivity but presents risks of toxicity and rhizosphere acidification

Yield outcomes in drip-NIs systems should be treated as an emergent consequence of (i) sustained availability of N in the root-active zone, (ii) the improvement in water status, and (iii) changes in the N form (NH_4_^+^ versus NO_3_^-^) experienced by roots over time. Drip fertigation is often reported to enhance yield and result in better water and nitrogen productivity (Tao et al., 2021b), and another study shows that such productivity can coincide with reduced nitrate leaching and reduced N_2_O at the system level, which is the “co-benefit” signature this review targets (Li et al., 2026).

From a plant-physiology perspective, maximizing ammonium retention is not always desirable. Under certain conditions, NH_4_^+^ may promote growth and reduce nitrate accumulation, yet continued NH_4_^+^-dominant nutrition may induce symptoms of toxicity or nutrient imbalances depending on the species, availability of counter-ions, and pH buffering with responses mediated by rhizosphere chemistry and plant metabolic capacity (Esteban et al., 2016; Marino and Moran, 2019). Tomato and cucumber are notable examples of ammonium-sensitive crops: prolonged NH_4_^+^ nutrition reduced tomato growth and yield through impaired Ca and Mg nutrition; similarly, cucumber exhibited suppressed growth and marked disruption of nitrogen and mineral metabolism (Roosta and Schjoerring, 2007; Borgognone et al., 2013). The real goal of drip-NIs integration is therefore not “maximum NH_4_^+^ availability” but “managed NH_4_^+^” during root uptake while preventing ammonium accumulation that may suppress root activity, micronutrient balance, and cation uptake. This risk is most likely under drip-NIs integration in the near-emitter core, where NH_4_^+^ concentrations are highest and rhizosphere acidification pressure may be greatest. Long-term nitrogen-driven soil acidification is a well-documented phenomenon in intensive croplands, and therefore multi-season experiments should track variations in pH over time (not merely short-term changes) and assesses the NH_4_^+^ benefits alongside acidification risk (Guo et al., 2010). In addition, we suggest that the crop responses should be interpreted in relation to known bulb conditions (WFPS/oxygen proxies, NH_4_^+^/NO_3_^-^ partitioning, rooting distribution), since the same applied fertilizer rate can lead to varying N form experienced by roots depending on changes in wetting geometry and pulse frequency.

### 6.3 Targeted nitrification inhibition restructures nitrogen cycling community

A scientific framing must not ignore the fact that inhibitors and irrigation do not simply change rates; they alter microbial selection pressures and spatial niches. Nitrification inhibitors tend to act more effectively on ammonia-oxidizing bacteria than on ammonia-oxidizing archaea, suggesting that their effectiveness may be dependent on which guild dominates ammonia oxidation in a particular soil and microenvironment (Lei et al., 2022). In drip systems, that guild structure may be spatially structured: repeated pulses could generate an “oxic front” and a wetter core, while the periphery may experience stronger drying and substrate limitation. This may make microbial inference from bulk soil less representative of conditions within the wetted bulb.

There is also evidence of non-target and network-level effects. For example, nitrapyrin has been shown to change the composition of the broader microbial community, and a variety of nitrogen-cycle genes, with effects varying over time and across compartments (Schmidt et al., 2022). This is important for drip-NIs designs that a targeted application can still restructure communities within the bulb, which may change the denitrification capacity (including N_2_O reduction potential) and change resilience to pulses (e.g., irrigation-induced redox oscillations). A more testable claim here is that community restructuring should be judged as a spatially resolved “functional shift”: (i) depressed amoA potential at the oxidation front, along with (ii) maintained or increased N_2_O reduction potential (nosZ-linked potential) in wetter microzones where denitrification is most likely to take place. The practical implication is that microbial claims should be supported by spatial sampling designs (rhizosphere vs bulk; core to periphery) and paired fate-function measures (where possible) (e.g., inhibitor residues and amoA, nirK/nirS, nosZ markers) rather than single-point bulk-soil assays.

### 6.4 Trade-offs and failure modes including salinity accumulation, pH drift, oxygen limitation, root stress, and off-target impacts

Trade-offs are not side notes in drip-NIs systems; they establish the boundary conditions of scalability (**Figure 5**). There are four failure modes that are common and deserve specific attention:

**Figure 5:**
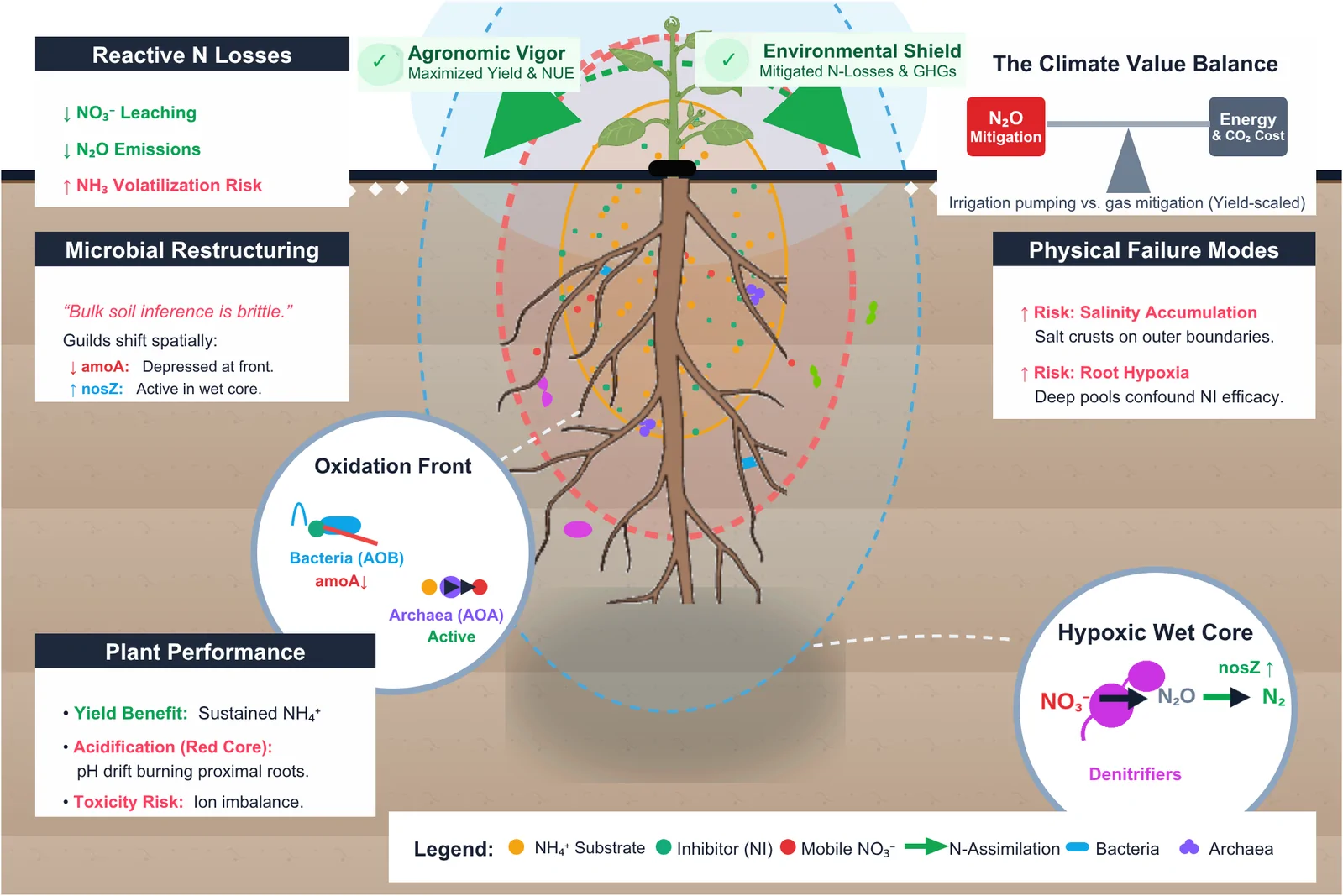
Whole-system conceptual overview of the mechanisms and biogeochemical trade-offs of drip-NIs integration. The diagram illustrates the potential benefits of co-localizing drip-NIs within the rhizosphere while simultaneously presenting the physiological and physical trade-offs.

1. Salt accumulation in localized wetting patterns. Drip localization (in particular under film mulch) may create salt redistribution, with desalination in shallow layers but accumulation in deep layers or wetting boundary during particular regimes. HYDRUS-directed analysis and field validation in an arid saline-alkali environment revealed notable differences between leaching areas and salt accumulation layers in film-mulched drip-irrigation (Li et al., 2023b). This is important for drip-NIs integration since any mechanism that alters root activity and uptake timing (through NH_4_^+^ retention) can indirectly shift the pattern of salt concentration by changing transpiration and root water extraction patterns.
2. pH drift and acidification. NIs delay and relocate the pattern of proton generation, but they do not eliminate acidification pressure if nitrification ultimately occurs (Li et al., 2023a). In intensive systems, cumulative N input has also been associated with significant soil acidification, making pH a slow variable that may reverse long-term benefits unless liming, buffering capacity, and base-cation dynamics are managed (Guo et al., 2010). In the DRM framework, pH must be considered a state variable rather than a soil background property that may vary across seasons and affect inhibitor performance and nutrient balance of crops.
3. Oxygen limitation and root stress. Drip has the ability to minimize extreme saturation compared with certain conventional irrigation methods; however, improperly designed schedules, emitter blockage or poor uniformity, or dense soils may sustain low-oxygen microsites. Roots under oxygen deprivation have diminished functionality and increased stress sensitivity (Salvatierra et al., 2020), and plant tolerance is determined by adaptive traits, including the formation of aerenchyma and metabolic adjustments (Colmer and Voesenek, 2009). More importantly, persistent low O_2_ may be a confounding factor: it may inhibit nitrification regardless of inhibitor presence, causing apparent NI efficacy to be high even when inhibitor-microbe contact is limited.
4. Off-target impacts and environmental fate. NI adoption increasingly depends on fate, risk assessment and residues, rather than efficacy. A recent review paper synthesized exposure, degradation and transport pathways in the context of nitrification and urease inhibitors in soil and water, reinforcing the necessity to combine performance trials with risk and residue-based measurements where possible (Wang et al., 2025). Because localized high concentrations can occur under fertigation, therefore drip delivery can be an efficiency benefit as well as a potential hotspot risk if the residues persist or move off-site.

### 6.5 Net environmental performance including climate and water-quality co-benefits with uncertainty bounds

A credible net-benefit assessment must include direct soil emissions together with the carbon and energy costs of pressurized irrigation and pumping. A recent global assessment estimated the energy consumption and carbon dioxide (CO_2_) emissions associated with irrigation and identified groundwater pumping as the dominant driver of irrigation energy demand, implying that the climate value of N_2_O mitigation needs to be discussed together with irrigation energy intensity and regional power mixes (Qin et al., 2024).

In the context of the DRM framework proposed in this review, two reporting conventions have been used to make claims policy-relevant and comparable across studies: (i) yield-scaled measures (e.g., N_2_O per unit yield; nitrate leaching per unit yield) and (ii) uncertainty limits that indicate the variability across soils, climates, and drip configurations rather than individual site estimates. The synthesis is clear: drip-NIs systems have the potential to provide real co-benefits to water quality and greenhouse forcing; however, only if hydrologic design (pulsing and wetting geometry), inhibitor selection, and site constraints (buffering capacity, salinity risk, oxygen regime) are managed as a coupled design problem, rather than independent management levers (Kuang et al., 2021; Zheng et al., 2023; Qin et al., 2024).

## 7 Research roadmap for deployable rhizosphere control

As discussed in Section 5, we mentioned that drip systems can be considered as a repeatable wetted-bulb reactor and nitrification inhibitors may become more efficient when their delivery is synchronized with the location and timing of active ammonia oxidation. The next step is to make the idea practical: quantify the appropriate state variables within the bulb and relate them to in-soil process rates, and use that knowledge to guide fertigation and inhibitor dosing in ways that are consistent across drip designs, soils and climates. Finally, the decision-making framework presenting the NI formulation recommendations under targeted nitrogen-management scenarios is given in **Figure 6**. The framework emphasizes a context-specific approach to mitigate nitrogen losses in the rhizosphere. The practical implications are outlined below.

**Figure 6:**
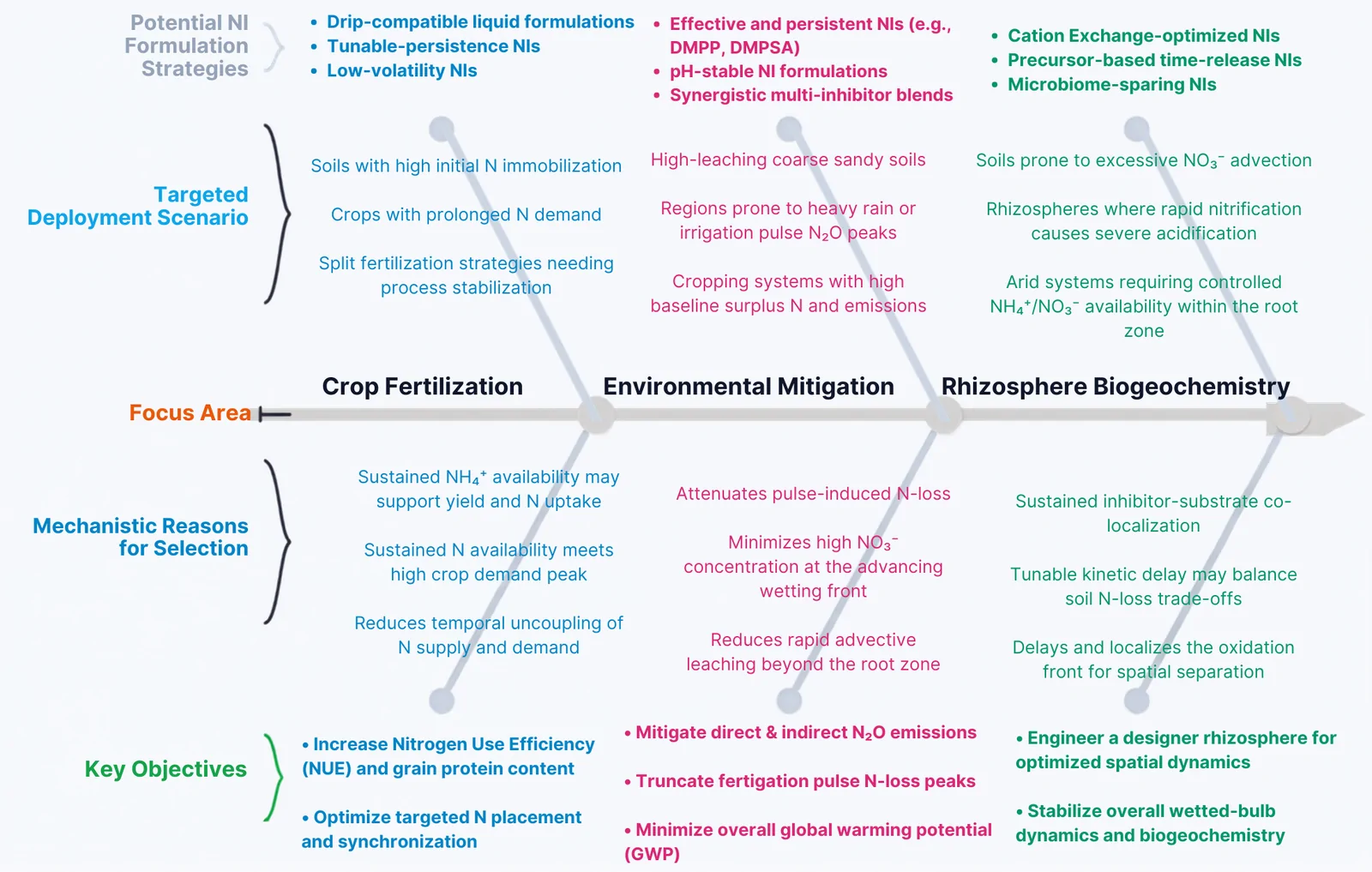
Purpose-specific drip-NIs integration; a conceptual guide for crop fertilization, nitrogen loss mitigation, and rhizosphere biogeochemistry.

### 7.1 Measurement priorities including oxygen microprofiles, *in situ* nitrification rates, isotope approaches, and functional gene activity

Most drip-NIs trials still rely on bulk soil sampling and end-of-season balances that fail to adequately resolve the processes influencing the occurrence of hot moments after irrigation pulses. The measurement focus must therefore shift from average concentrations to state variables that govern rates within the wetted bulb: WFPS, oxygen supply, ammonium and nitrate partitioning, and inhibitor exposure at the nitrification front.

The core validation experiment should therefore use a replicated factorial design that contrasts drip configuration or irrigation pulse regime, NI-treated and untreated fertilizer, and where possible, different soil textures. At spatially matched positions within the oxidation front, wetted-bulb core, and periphery, measurements should combine oxygen microprofiles, NH_4_^+^ and NO_3_^-^ distributions, inhibitor residues, composition and activity of ammonia-oxidizing communities, and isotopic ^15^N-based estimates of gross nitrogen transformations. Such an integrated design would investigate whether NI exposure coincides with active nitrification zones, which is a central assumption of the DRM.

A key upgrade is spatial explicitness. A drip-NIs application is not a uniform treatment: the bulb core, the oxic margin (oxidation front), and the periphery can act as different reactors within the same plot. Therefore, sampling designs should be aligned with these compartments rather than relying on arbitrary depth-only increments. In practice, the minimum mechanistic package in most field or greenhouse-soil studies can be stated up front: (i) volumetric soil water content (θ) or WFPS at several radii, and depths around the bulb, (ii) NH_4_^+^ and NO_3_^-^ profiles that are aligned to those points, (iii) the NI-exposure proxy (measured residues where possible, or at least timed dosing and hydraulic characterization), and (iv) event-resolved samples around irrigation pulses (before, immediately after, and during the oxic recovery window). This mechanistic package will make it possible to determine whether inhibition actually overlaps the zone in which nitrification was possible. Gross nitrification and mineralization may also be measured by ^15^N isotope pool dilution and tracer methods, which are designed to separate the production and consumption terms that would otherwise confound concentration-based interpretation.

Third, functional gene measurements are most useful when they are specifically related to microenvironmental gradients and to the timing of irrigation pulses. Many studies report gene abundances without locating them within the wetted-bulb structure. Thus, spatial sampling (core-front-periphery; rhizosphere vs bulk) is a more informative minimum package that should be combined with amoA (AOB and AOA), nirK/nirS, and nosZ, where feasible. Further, investigations on drip-fertilized maize demonstrate how nitrogen source and drip set-up can concurrently affect N_2_O emissions and N-cycling gene expression, offering a template for linking configuration, microenvironment, and microbial function (Guardia et al., 2024).

### 7.2 Practicing sensor-guided fertigation through nitrate and N_2_O feedback loops

A realistic control agenda does not require the existence of futuristic hardware: it demands closing the loop on measurable indicators that meaningfully constraint decisions. Credible online nitrate data are already available, showing that real-time soil nitrate information can be used to guide fertilization and these data can be translated into a practical approach rather than only *post hoc* diagnostics (Yekutiel et al., 2024). Simultaneously, *in situ* electrochemical soil nitrate sensing approaches are advancing toward field-deployable designs, which is important because drip systems require measurements at the bulb scale rather than at the field-average scale (Eldeeb et al., 2023).

In the context of irrigation, intelligent fertigation systems, which automate the delivery of water and nutrients, have proven to be agronomically feasible in high-value horticulture, with outcomes such as increased yield and quality, and reduced irrigation requirements in tomato (Wang et al., 2024). For drip-NIs integration, the near-term “feasible now” is not complete autonomy, but rather a simple multi-depth, multi-radius monitoring plan that tracks (i) soil moisture/WFPS and (ii) the dynamics of NO_3_^-^ at the oxidation front, such that the nitrate front can be maintained within the active root zone by adjusting the pulse frequency and NO_3_^-^ concentration.

The N_2_O feedback is more difficult, but is now becoming more plausible for research-to-deployment translation. Recent measurement methods seek to record event-resolved flux dynamics with lower power requirements and improved temporal resolution than traditional chamber sampling methods, which is important because irrigation-driven pulses can dominate seasonal totals (Brown et al., 2024). The portable laser spectroscopic systems also illustrate how more field-deployable measurements of higher frequencies can be made to provide better attribution of emissions to particular fertigation events (Stiefvater et al., 2023). In the next step, these tools may then be used to provide “learning control”, i.e., measured pulses can be used to update models and optimize dosing strategies even if real-time N_2_O control is not yet practical in most farms.

### 7.3 Designing next-generation inhibitors tuned to wetted-bulb chemistry

If drip fertigation creates a physically constrained reaction domain, then inhibitor design must explicitly target compatibility with it: it must be soluble in fertigation solutions, exhibit predictable mobility within the bulb, and persist at the right location according to irrigation waveforms.

There are two near-term priorities. First, the performance of inhibitors is highly dependent on soil chemistry, and pH effects can be substantial enough to alter the mitigation results. Recent findings on pH-sensitive performance reinforce that site chemistry is a part of the inhibitor-selection logic, rather than merely of *post hoc* explanation (Li et al., 2024). Second, wetting-drying history in drip systems is not merely a background condition; it determines both nitrification windows as well as inhibitor-exposure pathways. Experiments that directly examine the effects of drying-rewetting on DMPP and DMPSA demonstrate that pulse regimes may modify the behavior of different inhibitors and hence the practical duration of inhibition (Sánchez-Martin et al., 2024).

New inhibitory chemistries have also been discovered alongside optimization of existing molecules. For example, 4-methyl-1-(prop-2-yn-1-yl)-1H-1,2,3-triazole (MPT) has been reported as a strong nitrification inhibitor, which shows that the pipeline of “drip-compatible” inhibitors is real rather than speculative (Yildirim et al., 2024). Furthermore, for drip systems, the screening logic must also cover wetted-bulb transport behavior (retention vs bypass) and compatibility with typical fertigation chemistries rather than just inhibition potency.

### 7.4 Process-based models coupling water flow with microbially explicit nitrogen transformations

The future utility of the DRM will depend on whether experimentally validated relationships can subsequently be translated into predictive rules or not. This requires models that represent the wetted bulb as a three-dimensional transport and reaction domain, rather than a uniform soil layer.

Hydrological modeling frameworks already exist that can simulate drip wetting patterns and solute transport under localized irrigation. Specifically, the HYDRUS family of models is widely used for variably saturated flow and reactive transport problems relevant to fertigation (Šimůnek et al., 2016). Complementary process-based models include DayCent and DenNitrification-Decomposition model (DNDC), which simulate soil carbon and nitrogen transformations and associated N_2_O emissions, and Root Zone Water Quality Model 2 (RZWQM2) which couples crop growth with soil water and nitrogen dynamics, and can evaluate management effects on crop nitrogen uptake and nitrate leaching (Giltrap et al., 2010; Ma et al., 2012; Scheer et al., 2014). However, in their standard configurations, these models do not resolve the combined effects of three-dimensional wetted-bulb geometry, NI sorption, degradation, and redistribution. Thus, future model development should integrate the multidimensional transport capability of drip-irrigation models with biogeochemical and crop-growth components of these established frameworks.

The most useful decision support models may not simulate every process. They should target a limited number of outputs that directly translate into management decisions: the location of the oxidation front, the rate of nitrate front movement in a specified pulse regime, and the dose of an inhibitor required to maintain effective concentration levels during the post-irrigation oxic recovery phase. Sensor data streams such as NO_3_^-^ and moisture can be used to update state estimates and to decrease seasonal uncertainty (Yekutiel et al., 2024). In the long run, this is how the field can shift from “trial-and-error NI use” toward more reproducible, transport-informed dosing logic that can be compared across studies and transferred across sites.

Among these research directions, the immediate priority is direct experimental validation of the DRM. Sensor-guided management, inhibitor redesign, and process-based modeling should follow once the spatial and temporal relationships underlying the framework have been established under field conditions.

## 8 Conclusion

This review demonstrates that effectiveness of integrating drip irrigation with NIs not only depends on managing nitrogen inputs, but also on controlling when and where nitrification occurs within rhizosphere. Nitrogen transformation outcomes emerge from interactions among oxygen availability, soil hydraulic behavior, microbial activity and inhibitor persistence within root-zone environments. This perspective also helps to reconcile inconsistent findings reported across NI types and crops, indicating that variable responses may reflect differences in rhizosphere conditions rather than inhibitor chemistry alone. The synthesized literature further reveals that reductions in gaseous nitrogen losses and leaching under drip-NIs systems are not universal and depends on careful management of the hydrological and biogeochemical heterogeneity of drip irrigated soils. However, the DRM remains a conceptual framework that requires validation before it can be translated into practical management recommendations. Thus, future nitrogen management approaches should shift from generalized recommendations to context-specific designs adopted to local irrigation and soil conditions. Future research should therefore prioritize field validation of the DRM and long-term assessment of microbial responses, soil functioning, salinity, and acidification through integrated hydrological modeling, high-resolution rhizosphere measurements, and precision irrigation experiments across contrasting soils and climates. Such evidence is essential for translating drip-NIs integration into site-specific management recommendations that improve nitrogen retention while minimizing environmental losses.
